# On the Relevance of Mental Imagery Beyond Stress-Related Psychiatric Disorders

**DOI:** 10.3389/fpsyt.2014.00077

**Published:** 2014-07-03

**Authors:** Jan Philipp Klein, Steffen Moritz

**Affiliations:** ^1^Department of Psychiatry and Psychotherapy, University of Lübeck, Lübeck, Germany; ^2^Department of Psychiatry and Psychotherapy, University of Hamburg, Hamburg, Germany

**Keywords:** major depressive disorder, obsessive–compulsive disorder, mental imagery, hallucinations, cognition

If a patient with major depressive disorder (MDD) reported that he is hearing self-derogatory thoughts aloud he would probably be diagnosed with psychotic depression. This might be followed by a change in medication rather than the offer of specific therapeutic strategies that target intrusive mental images. Yet in support of a growing literature of mental imagery in psychiatric disorders (1), recent research by our group shows that about one in two patients with mild to moderate MDD and three in four patients with obsessive–compulsive disorder (OCD) report sensory properties of their cognitions (2, 3).

In fact, most of us share such experiences: if we remember how we met our loved one, we sometimes see a visual image of how we first met them and this visual image can be accompanied by intense positive emotions. Similarly, we may vividly remember how it hurt when we were beat up in the school yard and again this tactile image may come with intense negative emotions.

On a more systematic level, mental imagery has been defined as the experience of conscious contents that possesses sensory properties and therefore resembles actual perceptual experience (1, 4, 5). The perceptual properties can be visual but can also cover other sensory modalities such as tactile, acoustic, or somatic experience. In contrast to cognitions, mental images are not purely verbal or abstract (5).

If these mental images occur involuntarily they are also referred to as “intrusions” (5). Mental imagery has been recognized in a number of disorders (5). Intrusions are a diagnostic feature of stress-related disorders such as acute stress disorder, post-traumatic stress disorder (PTSD), and dissociative disorder (6). While intrusive mental images are also recognized in OCD, they are not part of the diagnostic criteria of depressive disorders (6).

Intrusive mental images can be regarded to exist on a spectrum where actual psychotic symptoms are at the far end and defined by impaired reality testing [Ref. (7), p. 371]. Interestingly, the definition of hallucinations [Ref. (6), p. 87] overlaps with that of intrusive mental images. Just like hallucinations, intrusive mental images may at times be vivid and clear, have the full force and impact of normal perceptions and be out of voluntary control (8).

To illustrate this point, it has been found by our group that a considerable number of healthy controls and patients with OCD also report hearing voices (8). The same study showed that only about a third of schizophrenics (31.1%) but a full-third of healthy “voice-hearers” (33.3%) experience their voices as not distinguishable from real voices. One distinguishing factor between acoustic mental imagery and hallucinations that emerged from that study was the conformity with personality: while two-thirds (66.7%) of healthy voice-hearers reported that their voices reflected their inner thoughts, only 15.6% of schizophrenics experienced it this way. Schizophrenics often describe their voice as someone else talking to them.

These findings can be interpreted in the context of a growing body of evidence that hallucinatory experience may not be confined to psychotic disorders as previously thought. In one recent meta-analysis, the annual incidence of psychotic experiences was reported to be as high as 2.5%, the meta-analysis also found that <10% of patients reporting psychotic experiences actually go on to develop a psychotic disorder (9).

In conclusion, there is growing evidence that intrusive mental images are probably present in a wide range of mental disorders and lie on a continuum that ends in frank hallucinatory experiences (1, 5). To complement and expand the knowledge of mental imagery in mental disorders, we have recently completed two online studies that tapped into sensory properties of cognitions in MDD and OCD.

One study was conducted as part of large randomized trial (10). Here, we examined to what extent depressive thoughts are accompanied by mental imagery and how this is associated with symptom severity, insight, and quality of life (2). We recruited a large sample of mildly to moderately depressed patients (*N* = 356) from multiple sources and asked them about sensory properties they may have experienced in association with their depressive thoughts and ruminations. Answers were collected on a five-point Likert scale (from none to extreme sensory experiences) on separate scales for each modality: visual, auditory, tactile (touch), somatic (i.e., bodily sensations), olfactory (smell), and other. We provided examples to illustrate possible sensory properties of thoughts. For instance, an auditory sensation would be that one perceives an “inner critic” who seems to have an actual voice and may call one a “loser.”

A total of 56.5% of our sample reported mental imagery in at least one sensory modality that was associated with their cognitions. The highest prevalence was seen for somatic (39.6%) followed by auditory (30.6%) and visual (27.2%) sensations. Strong or extreme sensory properties were reported by 10.7% for bodily, 5.6% for auditory, and 4.8% for visual sensations. Patients reporting sensory properties of thoughts showed more severe symptoms on the quick inventory of depressive symptoms (QIDS) and had more previous depressive episodes and were more often hospitalized in the past than those who did not.

In a separate study, we examined the prevalence of mental imagery associated with obsessions and its relationship with illness insight and depression (3). Here, we examined just 26 patients with OCD whose diagnosis was confirmed on a structured diagnostic interview that was conducted over the telephone. They were asked to indicate on the same scale as above if their obsessions were associated with perceptual features. Again, examples were given such as the tactile sensation of dirt on the skin in patients with washing compulsions.

A total of 73% of patients reported at least mildly perceptual features that were associated with their obsessions. Here, the predominant perceptual channels were somatic (54%, strong or extreme: 23%), visual (46%, strong or extreme: 19%), and tactile (35%, strong or extreme: 4%); only 12% reported that their obsessive thoughts hat acoustic properties (strong or extreme: 4%). The extent of perceptuality strongly correlated with lack of insight item on the Yale Brown Obsessive–Compulsive Scale (YBOCS) but only mildly with depression severity on the Beck Depression Inventory (BDI).

In summary, about one in two patients with mild to moderate MDD and a full three in four patients with OCD report mental imagery associated with their cognitions. The proportion may be even higher in severe depression; this is the subject of a currently ongoing study. The most common perceptual channels in both disorders are bodily and visual sensations; auditory sensations were common in MDD but not in OCD and tactile sensations were common in OCD but not in MDD.

One important limitation in these studies is that they rely on patients self-report regarding the sensory properties of cognitions. If confirmed in further studies, our findings could support the notion that we should recognize the presence of mental imagery in mental disorders other than just stress-related disorders. This may have implications for the understanding and treatment of these disorders. It has repeatedly been shown that interventions primarily targeting the verbal cognitive content do not significantly increase the effectiveness of cognitive behavioral therapy (3, 11). In spite of this, research of most mental disorders has focused on cognitive contents rather than other aspects of content such as mental imagery (5). Still, theories and therapies have been developed that go beyond targeting cognitive content (12). These include the theory that metacognitive beliefs (i.e., beliefs about the significance of cognitions: “If I think long enough I will find out what is wrong with me.”) rather than the cognitive content *per se* has negative consequences for self-regulation [Ref. (13), p. 232].

The current research presented here adds to this literature and suggests that in addition to cognitive content and metacognitive beliefs, mental imagery associated with cognitions may also impact the onset and maintenance of disorders such as depression and OCD. In support of this notion, a recent review came to the conclusion that mental imagery can contribute to the onset and maintenance of mental disorders as imagery invokes greater emotional responses than the verbal representation of similar events (1).

This may also have consequences for the treatment of these disorders (14), especially for those patients who are troubled by vivid and disturbing mental imagery. Our study suggests that this may be a considerable number of patients. We found that about 1 in 5 patients with OCD and 1 in 10 patients with mild to moderate MDD report strong to extreme mental imagery associated with their cognitions. Therapeutic techniques have been developed that aid in rescripting distressing imagery by having the patients focus on the intrusive sensory experience (most commonly visual experiences) and aiding them in vividly constructing an alternative outcome (5, 14).

## Conflict of Interest Statement

Jan Philipp Klein declares that he has received payments for presentations and workshops on psychotherapy for chronic depression and on psychiatric emergencies. These payments were made by the participants or their employer and not by any third parties. Steffen Moritz declares no conflicts of interest.
